# Antimicrobial Potential, Antioxidant Activity, and Phenolic Content of Grape Seed Extracts from Four Grape Varieties

**DOI:** 10.3390/microorganisms11020395

**Published:** 2023-02-03

**Authors:** Dimitrina Krasteva, Yavor Ivanov, Zlatina Chengolova, Tzonka Godjevargova

**Affiliations:** Department Biotechnology, University “Prof. Dr. A. Zlatarov”, 8010 Burgas, Bulgaria

**Keywords:** antimicrobials, grape seeds, composition, antioxidant activity, antimicrobial potency, pathogenic bacteria

## Abstract

The aim of this study was to evaluate the total phenolic content, composition, and antioxidant and antibacterial activities of four grape seed extracts (Cabernet Sauvignon, Marselan, Pinot Noir, and Tamyanka). The total phenolic content (TPC) and flavonoid, anthocyanin, procyanidin, ascorbic acid, DPPH, and ABTS antioxidant capacities of the grape seed extracts (GSEs) were determined. The extracts showed high TPC values (79.06–111.22 mg GAE/g). The individual components in the GSEs were determined using HPLC. High contents of catechin, epicatechin, and procyanidin B1 were found in the extracts. The antimicrobial activity of the obtained GSEs against *Staphylococcus aureus*, *Bacillus cereus*, and *Escherichia coli* was evaluated using the agar diffusion test and a test to determine the minimum inhibitory concentration (MIC). According to the effect on the growth of pathogens, the extracts were ranked in the following order: Pinot Noir > Marselan > Cabernet Sauvignon > Tamyanka. The tested bacteria showed high sensitivity to the extracts (MIC = 0.12–0.50 mg/mL). According to the MIC values, the bacteria were in the following order: *S. aureus* > *B. cereus > E. coli*. A correlation was found between the phenolic content of the GSEs and their antibacterial potential. The obtained results show that the studied GSEs have good potential as antioxidant and antimicrobial agents.

## 1. Introduction

Ensuring food safety is an important task around the world. Foods containing pathogenic bacteria, such as *Escherichia coli*, *Staphylococcus aureus*, and *Bacillus cereus*, are very dangerous for human health [1]. Currently, synthetic chemical preservatives are mainly used as antimicrobial agents in foods, but they show cytotoxic effects [2]. Research on the application of plant extracts, especially grape seed extracts, as alternative antioxidants and antibacterial agents in foods is constantly expanding [3,4,5]. The polyphenols contained in red grapes, especially in grape seeds and skins, have high antioxidant capacity and high antimicrobial and antilipid potencies [6,7]. The largest proportion of polyphenols (60–70%) is concentrated in grape seeds [8]. In recent years, these compounds have been extracted from grape seeds and used as food additives, antimicrobial agents, and inhibitors of dietary lipid oxidation [9].

The antimicrobial activity of compounds recovered from grape seed extract (GSE) has not been widely studied [10,11]. There is little information about the relationship between the polyphenolic compounds in GSEs and their antimicrobial activity [12,13]. Some authors have investigated the antibacterial activity of pure phenolic compounds. They have reported that the chemical structure of phenolic compounds, the number of hydroxyl groups, and the degree of their polymerization influence their antimicrobial activity [14,15]. Khameneh et al. (2019) [16] reported that phenolic compounds showed different mechanisms of action against different bacterial strains. Grape seed extract contains phenolic compounds that may act through various mechanisms, thus preventing bacteria from becoming completely resistant. Phenolic extracts have shown higher antimicrobial potency than pure phenolic compounds [17]. The reason for this may be that there is a synergistic effect between the components of the extracts [18]. This is why it is important to study the phenolic content and composition of GSEs and their correlation with antimicrobial activity. There are many publications about the composition of polyphenolic compounds in red grape seed extract and only a few studies on white grape seed extract [19,20]. The reason for this is that red grape seed extract has higher polyphenolic content than white grape seed extract [11,21].

Complete characterization of the polyphenolic content of GSEs is carried out by determining indicators, such as total phenolic content (TPC), total flavonoids (TF), total procyanidins (PC), and total anthocyanins (TA), using spectrophotometric methods [22,23]. An important indicator for characterizing GSEs is their antioxidant capacity, which is measured using different methods, including DPPH, ABTS, and ferric reducing power [24,25]. Researchers have found a good relationship between total phenolic indicators and antioxidant activity [26,27,28,29]. In many publications, the individual compounds contained in GSEs were analyzed using HPLC [7,12,19,27,28,29,30]. Some have indicated that the main compounds in GSEs are the following: gallic acid, (+)-catechin, (−)-epicatechin, (−)-epigallocatechin gallate, dimeric and trimeric procyanidins, and trans-resveratrol. It has been reported that the composition profile is responsible for the antioxidant and antibacterial activities of GSEs [7,21,29].

Some authors have reported a correlation between the total phenolic indicators of GSEs (TPC, TF, and PC) and their antioxidant and antimicrobial capacities [13,31], but there is little information on the connection between the composition profile of GSEs determined with HPLC and their antimicrobial activity [12,32]. All publications have shown that GSEs have the potential to be used as natural antimicrobials in food products.

The aim of this study was to evaluate the phenolic content and the antioxidant and antimicrobial potencies of GSEs from *Vitis vinifera* L. cv. Cabernet Sauvignon, Marselan, Pinot Noir, and Tamyanka grapes grown in the same geographical area. Characterization of the extracts was carried out to better understand the compounds present in them that contribute to antioxidant and antimicrobial activity. The extracts were analyzed using a number of spectrophotometric methods to determine the antioxidant capacities of TPC, TF, PC, TA, DPPH, and ABTS and by using HPLC to determine the individual compounds. Antimicrobial testing to establish the minimum inhibition activity of the extracts was performed using *B. cereus*, *E. coli*, and *S. aureus*. The objectives of this study were to (1) determine the phenolic content and composition of GSEs from four grape varieties and (2) evaluate their antioxidant and antimicrobial activities to determine their potential as sources of natural antioxidant and antimicrobial agents.

## 2. Materials and Methods

### 2.1. Materials and Chemicals

The materials used in this study consisted of waste (pomace) from the vinification of red wines from *Vitis vinifera* L. cv. Pinot Noir, Marselan, and Cabernet Sauvignon grapes and white wine from *Vitis vinifera* L. cv Tamyanka grapes (Pink Pelikan Winery Ltd, Silistra, Bulgaria). Marselan is a recent crossing between two famous red grape varieties, *Vitis vinifera* L. cv. Grenache and Cabernet Sauvignon. Tamyanka is a French Muscat. These grape varieties are grown in the Danube region near the city of Ruse, Bulgaria. 

The microorganism strains *S. aureus* (35548), *E. coli* (10536), and *B. cereus* (33019) were provided by the American Type Culture Collection and stored at the Department of Biotechnology, University-Burgas.

The chemicals used for the experiment were ethanol 99.9% *v*/*v* (Valerus, Sofia, Bulgaria), acetonitrile 100% (HPLC grade), phosphoric acid, sodium carbonate (>99%), 2,2-diphenyl-1-(2,4,6-trinitrophenyl)-hydrazinyl (DPPH), 2,2-azino-bis (3-ethylbenzothiazoline-6-sulphonic acid) diammonium salt (ABTS), 6-hydroxy-2,5,7,8-tetramethylchroman-2-carboxylic acid (Trolox 97%), 2N solution of Folin–Ciocalteu reagent, and sodium hydroxide, purchased from Sigma-Aldrich, Darmstadt, Germany. The reagents sodium nitrite and aluminum chloride hexahydrate were purchased from Merck, Germany. For HPLC analysis, the following standards were used: gallic acid–anhydrous, quercetin hydrate, (+)-catechin hydrate, (−)-epicatechin, procyanidin B_1_, procyanidin B_2_, procyanidin B_3_, procyanidin C_1,_ rutin, gallic acid glucoside, resveratrol, and (−)-epigallocatechin gallate (Sigma Aldrich, Darmstadt, Germany). Deionized water purified by an ELGA water purification system (UK) was used throughout the experiment.

### 2.2. Production of Grape Seed Powder

The seeds were separated from the skin using a plastic sieve. Then, the treatment of the grape seeds was carried out separately, with processing by washing and drying at 40 °C for 14 h and storage at 4 °C. For each experiment, a certain quantity of dry grape seeds was ground to powder with a diameter of 2.5–22.5 μm.

### 2.3. Preparation of Grape Seed Extract

A mixture of grape seed powder (5 g) with 25 mL of 70% aqueous ethanol was stirred using an MMS-3000 magnetic stirrer (Boeco, Hamburg, Germany) at a constant rate of 500 rpm at ambient temperature and pressure for 3 h. The upper liquid was separated, and the process was repeated with another 25 mL of 70% aqueous ethanol. The resulting mixture was centrifuged at 6000 rpm for 10 min. The supernatant was separated and concentrated to 1 mL in a vacuum evaporator (Rotavapor R-215, Buchi, Flawil, Switzerland) in a water bath at 50–60 °C and 100–175 hPa vacuum pressure.

### 2.4. Spectrophotometric Analysis

#### 2.4.1. Total Polyphenolic Content

TPC was determined using the Folin–Ciocalteu assay [33]. Absorbance was measured at 725 nm on a Jenway 6900 UV-Vis spectrophotometer (Colmworth, UK). To 50 µL of GSE, 3 µL of ultrapure water and 50 µL of 2N Folin–Ciocalteu reagent were added. The mixture was left in the dark for 3 min and then 300 µL Na_2_CO_3_ (75 g/L) was added. The obtained mixture was incubated in the dark at room temperature for 1 h. Absorbance was measured at 725 nm on the Jenway 6900 spectrophotometer. The standard curve was obtained based on gallic acid with a concentration of 50–450 µg/mL in ethanol. TPC was calculated as milligram equivalent gallic acid per gram of dried matter (mg GAE/g DW). According to the literature, the incubation time should be 1 h [34] or 1.5 h [35]. After the experiments, it was concluded that 1.0 h was sufficient to obtain the proper results.

#### 2.4.2. Total Flavonoids

TF was determined using an aluminum complexation assay [23]. The tested extract (250 µL) was mixed with 1 mL ultrapure water and 75 µL 5% NaNO_2_ for 5 min. Then, 150 µL of a 2% aqueous solution of aluminum chloride was added. The sample was mixed for 6 min. After that, 500 µL 1N sodium hydroxide was added, and the sample was incubated in the dark at room temperature for 11 min. The samples were diluted with 4 mL d.H_2_O. The formed complex was analyzed at 510 nm on the Jenway 6900 UV-Vis spectrophotometer. The standard curve was obtained based on a methanol solution of quercetin with a concentration of 0–1 mg/mL. Total flavonoids were expressed as milligram equivalent quercetin per gram of dried matter (mg QE/g DW).

#### 2.4.3. Procyanidins

PC was determined using the method developed by Sun et al. (1998) [36]. A GSE solution with a concentration of 300 μg/mL in methanol was prepared. A calibration curve was prepared using absorption (A) for catechin (CT) solutions with concentrations ranging from 20 to 500 μg/mL. Different mixtures were prepared and incubated for 20 min at 30 °C and then their absorption was measured at 500 nm using the Jenway 6900 spectrophotometer. The mixtures were as follows:

A_0_ = [1 mL methanol + 2.5 mL methanol + 2.5 mL 9 mol/L HCl]
A_b_ = [1 mL methanol + 2.5 mL 1% vanillin solution + 2.5 mL 9 mol/L HCl]
A_c_ = [1 mL CT (20–500 μg/mL) or GSE + 2.5 mL methanol + 2.5 mL 9 mol/L HCl]
A_s_ = [1 mL CT (20–500 μg/mL) or GSE + 2.5 mL 1% vanillin + 2.5 mL 9 mol/L HCl]
Absorption was calculated for each standard and sample solution as follows: A = (A_s_–A_b_)–(A_c_–A_0_).

The total procyanidin content in each test solution was calculated from the calibration curve and expressed as mg (+)-catechin per gram of dried matter (mg CE/g DW). 

#### 2.4.4. Total Anthocyanins 

TA content was analyzed according to the methodology described by Lee et al. (2005) [37]. The extract (250 µL) was diluted with buffer solution at pH 1 to obtain an absorbance of 1.1. The dilution factor was determined. Then, the extract was diluted with buffer solution at pH 4.5 using the same dilution factor. The absorbance was measured at 520 and 700 nm on the Jenway 6900 UV-Vis spectrophotometer. Absorbance at 700 nm was measured due to the presence of turbidity in the solution. The concentration of anthocyanin pigments was expressed as cyanidin-3-glucoside equivalent (mg/L) and was determined using the following equation:

AC pigments = A · M · F · 10^3^/ε · 1, mg/L

A = (A_520nm_ − A_700nm_) _pH 1_ − (A_520nm_ − A_700nm_) _pH 4.5_

where M is the molecular weight of cyanidin-3-glucoside (449.2 g/mol); F is the dilution factor; 1 is the path length in cm; ε is the 26,900 molar extinction coefficient in L/mol·cm for cyanidin-3-glucoside; and 10^3^ is the factor for converting from g to mg.

#### 2.4.5. Ascorbic Acid Content

Ascorbic acid content was assessed according to the procedure described by Negro et al. (2003) [38]. Extract (500 µL) was mixed with 250 µL Folin–Ciocalteu reagent that was previously diluted with 4.5 mL ultrapure water. The mixture was thoroughly homogenized for 3 min at room temperature. If the solution was excessively cloudy, centrifugation or filtration was performed before the measurement. The solution absorbance was measured at 765 nm on the Jenway 6900 spectrophotometer. The ascorbic acid content was assessed based on the calibration curve of ascorbic acid (10–500 μg/mL in ethanol).

#### 2.4.6. Determination of ABTS Radical Cation Scavenging Activity

The ABTS assay was performed using the methodology described by Re et al. (1999) [39]. Equivalent amounts of 2.6 mM potassium persulfate and ABTS (7.4 mM in deionized water) were mixed and incubated in the dark at room temperature for 12–16 h. Fresh ABTS solution was prepared for each analysis. Before use, 1 mL ABTS+• solution was diluted with 60 mL methanol to obtain an absorbance of 1.1 at 734 nm using the Jenway 6900 spectrometer. Samples of GSEs (250 µL) at a concentration of 10 mg/mL were reacted with 2 mL of ABTS+• solution for 10 min in the dark. Then, the absorbance of the solution was measured at 734 nm with the spectrophotometer. A standard curve was drawn in the linear interval of 25–600 µM Trolox. The results are expressed as µM Trolox equivalent per gram dry weight.

#### 2.4.7. Determination of DPPH Radical Scavenging Activity

The DPPH assay was conducted using the method described by Brand-Williams et al. (1995) [40] and modified by Miliauskas et al. (2004) [41]. GSE samples (100 µL) at a concentration of 10 mg/mL were mixed with 2 mL of daily prepared DPPH• solution (24 mg in 100 mL methanol) and incubated for 20 min at room temperature. Then, the absorbance of the solution was measured at 515 nm on the Jenway 6900 spectrophotometer. A calibration curve based on Trolox standards (25–800 µM) was constructed. The results are expressed as µM Trolox equivalent per gram dry weight.

### 2.5. HPLC Analysis

HPLC analysis was performed on a Varian 920-LC Liquid Chromatograph, Sudbury, UK equipped with a Zorbax SB-C18 Stable Bond analytical column (5 μm, 4.6 mm × 250 mm), a Zorbax SB-C18 analytical guard column (5 μm, 4.6 mm × 12.5 mm), and a photodiode array (PDA) detector. The absorbance was read at 280 and 360 nm, and the PDA range was 200–600 nm. The column temperature was kept at 24 °C, and the injection volume was 10 μL. Mobile phases were used in gradient mode. The elution was performed using solvent A, 0.1% phosphoric acid in deionized water, and solvent B, acetonitrile. The mobile phases were filtered through a 0.45 μm filter and degassed before use. The gradient elution program was set as described in Table 1. The flow rate was 0.7 mL/min.

Dried extracts and standards were dissolved in 70:30 acetonitrile and deionized water. The solutions were filtered in a 0.8 μm filter unit and then a 0.45 μm filter unit before analysis. The stock standard solutions for the GSE assay, at a concentration of 1 mg/mL, were as follows: gallic acid, gallic acid glucoside, (+)-catechin, (−)-epicatechin, (−)-epigallocatechin gallate, procyanidin B_1_, procyanidin B_2_, procyanidin B_3_, procyanidin C_1_, rutin, quercetin, gallic acid glucoside, and resveratrol.

### 2.6. Preparation of Bacterial Inoculum

Inocula of the following microorganism strains were prepared: *S. aureus* (ATCC 35548), *E. coli* (ATCC 10536), and *B. cereus* (ATCC 33019). Using an inoculating loop, cells of the microorganisms were introduced into 3 mL of nutrient broth and incubated overnight on a shaker at 220 rpm at 37 °C. After that, the optical density (OD_650 nm_) of the bacterial solution was measured at 650 nm on the Jenway 6900 spectrophotometer. The obtained bacterial suspensions were diluted with nutrient broth liquid medium to obtain a suspension with OD_650nm_ = 0.3–0.5, corresponding to a cell concentration of 5 × 10^5^ CFU/mL.

### 2.7. Determination of Antimicrobial Activity Using the Agar Diffusion Method 

The antibacterial activity of the GSEs was determined using the agar diffusion method [42]. Bacterial strains were first grown on Mueller–Hinton medium in Petri dishes for 18–24 h at 37 °C. Sterile 6 mm diameter filter discs dipped in GSEs at different concentrations (0.50, 0.25, 0.10, and 0.05 mg/mL) were placed on the infusion agar seeded with bacteria. For the negative control, a disc impregnated with 0.9% NaCl without GSE was used. The antibiotic ciprofloxacin (25, 6.25, and 1.55 mg/L) was used as the positive control on a separate Petri dish. All Petri dishes were kept at 4 °C for 30 min and then incubated under aerobic conditions at 37 °C for 24 h. Antibacterial activity was assessed by measuring the zone of growth inhibition surrounding the filter discs. 

### 2.8. Minimum Inhibitory Concentration Test 

Minimum inhibitory concentrations (MICs) were determined using the method described by Das et al. (2021) [43]. The microbial inoculum was diluted to obtain a suspension with OD_650 nm_ = 0.3–0.5, corresponding to a cell concentration of 5 × 10^5^ CFU/mL, as described previously. Various concentrations of the extracts (6.00, 4.00, 3.00, 2.00, 1.50, 1.00, 0.50, and 0.25 mg dry weight per mL) were prepared. Then, 2 mL of each extract was added to 6 mL of diluted inoculum in tubes. The cultures were incubated at 37 °C for 48 h. The final concentrations of extracts in the tubes were 1.50, 1.00, 0.75, 0.50, 0.37, 0.25, 0.12, and 0.06 mg dry weight per mL. The growth of cells in the tubes was monitored by measuring the absorbance at 650 nm at 0, 5, and 24 h on the Jenway 6900 spectrophotometer. MIC was defined as the lowest concentration of GSE that inhibited visible bacterial growth after incubation for 24 h at 37 °C. Ciprofloxacin was used as the positive control, and the extract was in concentrations from 0 to 150 mg/mL.

### 2.9. Statistical Analysis

The experiments were performed in triplicate. The results are expressed as mean values ± standard deviation (SD). Analysis of variance (ANOVA) was performed (DPS 7.55 for Windows). 

## 3. Results

### 3.1. Chemical Composition and Antioxidant Activity of Grape Seed Extracts

The antimicrobial and antioxidant potentials of extracts from four varieties of grape seeds were investigated. First, the extracts were obtained and completely characterized in terms of total polyphenolic parameters, antioxidant capacity, and individual composition. Extracts were obtained from the seeds of four types of grapes (three red varieties, Cabernet Sauvignon, Marselan, and Pinot Noir, and one white variety, Tamyanka) using the following process: using an extracting agent, which was a mixture of ethanol and water (70:30), the mixture was stirred with a magnetic stirrer for 3 h at room temperature. It is well known that when using extracts in the food industry and in medicine, it is preferable to use ethanol as an extracting agent, since it is safer for health [12]. The specified extraction conditions were optimal, as experimentally determined. The extract yield was determined for all seeds. The lowest extract yield was obtained from the seeds of Pinot Noir grapes (12%) and the highest from the seeds of Marselan grapes (18%). The extract yields of the seeds from Cabernet Sauvignon and Tamyanka were 16 and 15%, respectively. 

In order to investigate the relationship between the total polyphenolic parameters of the extracts and their antimicrobial and antioxidant activities, the total polyphenolic content (TPC), total flavonoids (TF), procyanidins (PC), total anthocyanins (TA), and ascorbic acid (AA) values were determined using spectrophotometric methods (Table 2). 

TPC was found to be the highest in the Pinot Noir extract. According to TPC values, the extracts were ranked in the following order: Pinot Noir > Marselan > Cabernet Sauvignon > Tamyanka. TF and PC were the highest in the extracts of Marselan and Pinot Noir grape seeds. According to the TF values, the extracts were in the following order: Marselan = Pinot Noir > Cabernet Sauvignon > Tamyanka. According to the PC values, the order was: Marselan > Pinot Noir > Cabernet Sauvignon > Tamyanka. AA was present in all GSEs. The highest AA content was found in the Pinot Noir GSE. The anthocyanin pigment content was the highest in the Marselan and Cabernet Sauvignon GSEs. Anthocyanin pigments are found mainly in the skins of red grapes. The seeds should not contain anthocyanins, but traces of them usually remain when the seeds are separated from the skins, which is how they determine the color of the extracts. Color measurement of the GSEs was performed using a CR-410 Chroma Meter (Konica Minolta Co., Osaka, Japan) with a 5 mm diameter measuring area and a data processor (DP-301). The Chroma Meter was calibrated to the CIE color space system using a white tile. The three-color parameters of the GSEs were determined as follows: lightness L* = 41.84; redness a* = 4.21; yellowness b* = 21.78. Marselan GSE was found to have the highest redness value (14.10), followed by Cabernet Sauvignon (13.51). The color of Pinot Noir had a low redness value (4.21), which was almost equal to that of Tamyanka (3.95).

The antioxidant capacities of the four extracts were determined using two methods, DPPH and ABTS. These values are listed in Table 2. The Pinot Noir and Marselan GSEs had similar DPPH antioxidant capacities, which were higher than the other two types of extracts. The DPPH capacity of the Tamyanka white grape extract was the lowest. The results for ABTS antioxidant capacity were different. In this case, the ABTS antioxidant capacities of Marselan, Cabernet Sauvignon, and Pinot Noir were slightly higher than that of Tamyanka. These differences can be explained by the different mechanisms used to determine antioxidant capacity [39,40]. 

The individual components in the GSEs were determined by HPLC. Under the described HPLC conditions, the components were eluted in the following order: gallic acid, gallic acid glucoside, procyanidin-B_3_, procyanidin-B_1_, (+)-catechin, procyanidin B_2_, (−)-epicatechin, and procyanidin C_1_ (see Supplementary File). The concentration of resveratrol was very low. Epigallocatechin gallate, quercetin, and rutin were not detected in the extracts. The concentrations of individual compounds contained in the four extracts, determined by HPLC, are given in Table 3.

From Table 3, it is obvious that the Pinot Noir extract contained the highest amount of (+)-catechin (12.16 mg/g), and the Marselan extract contained the highest amount of (−)-epicatechin (14.33 mg/g). Procyanidin B_1_ also showed high concentrations and was most abundant in the GSEs of Pinot Noir and Cabernet Sauvignon.

Table 4 gives the total values of monomeric compounds (+)-catechin and (−)-epicatechin; dimeric compounds procyanidins B_1_, B_2_, and B_3;_ trimeric compound procyanidin C_1_; and TPC. It is obvious that the monomeric compounds were highest in the GSEs of Pinot Noir and Marselan. According to this indicator, the extracts are arranged in the following order: Pinot Noir = Marselan > Cabernet Sauvignon > Tamyanka. The sum of procyanidins B_1_, B_2_, and B_3_ values was highest in Pinot Noir, followed by Cabernet Sauvignon and Marselan.

As determined by HPLC, Pinot Noir GSE had the highest TPC; the extracts were ranked in the following order: Pinot Noir > Marselan > Cabernet Sauvignon > Tamyanka. When comparing the results obtained by the chromatographic and spectrophotometric methods, it can be seen that there is very good correlation. From all the obtained results, it can be concluded that the Pinot Noir and Marselan GSEs had the highest polyphenol content and antioxidant capacity.

### 3.2. Antibacterial Activity of the Different GSEs

**Figure 1:**
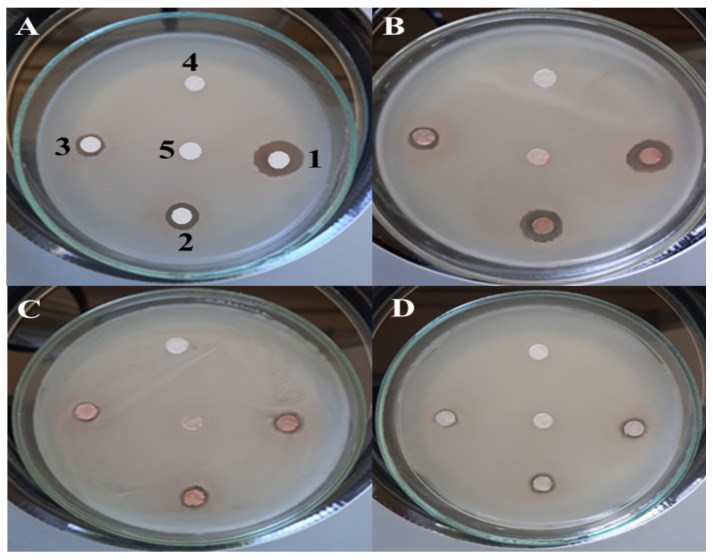
Antimicrobial activity of grape seed extracts against *S. aureus* (zone 1, 0.50 mg/mL extract; zone 2, 0.25 mg/mL; zone 3, 0.10 mg/mL; zone 4, 0.05 mg/mL; zone 5, negative control: (**A**) Pinot Noir, (**B**) Marselan, (**C**) Cabernet Sauvignon, and (**D**) Tamyanka.

The characterized extracts were tested for their antimicrobial activity against two Gram-positive bacterial species (*S. aureus* and *B. cereus*) and one Gram-negative bacterial species (*E. coli*). For this purpose, two methods were used: agar diffusion and a test for determining the minimum inhibitory concentration (MIC). The influence of the GSEs in different concentrations on the growth of *S. aureus*, *B. cereus*, and *E. coli* was studied. The results for *S. aureus* are shown in Figure 1; there are no pictures of the other bacteria because the results were similar. The antibiotic ciprofloxacin (25, 6.25, and 1.55 mg/L), as the positive control, was tested on a separate Petri dish.

The diameters of the inhibition zones (in mm) corresponding to the tested GSEs (0.50 mg/mL) and the antibiotic (25 mg/L) are listed in Table 5. All assays were carried out in triplicate. The results are expressed as mean ± SD.

The most sensitive strain to the extracts was *S. aureus*, and the least sensitive strain was *E. coli*. Higher concentrations of the extracts produced stronger inhibition. As described above, the extracts of Marselan and Cabernet Sauvignon had an intense color, and the areas formed by them were colored, especially at higher concentrations (Figure 1). The results show that the Pinot Noir and Marselan extracts had greater antimicrobial potential compared to the other two extracts (Table 5). The antibiotic ciprofloxacin with a concentration of 25 mg/L (positive control) showed higher antimicrobial activity than the GSEs against *S. aureus* (23 ± 0.33 mm), *B. cereus* (12 ± 0.15 mm), and *E. coli* (9 ± 0.11 mm).

**Figure 2:**
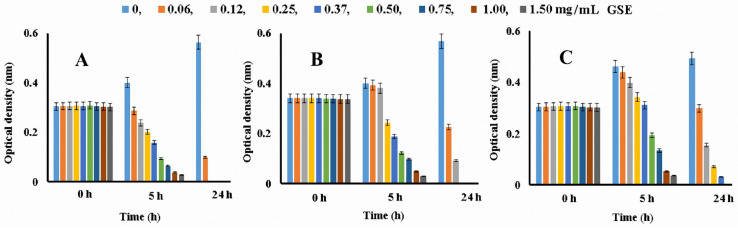
Quantitative evaluation of antibacterial activity of Pinot Noir GSE at different concentrations against (**A**) *S. aureus*, (**B**) *B. cereus*, and (**C**) *E. coli* during incubation at 0, 5, and 24 h (*n* = 5).

The MICs of the tested extracts were studied. The growth of *S. aureus* treated with different concentrations of Pinot Noir GSE at 0, 5, and 24 h is presented in Figure 2. From the figure, it can be seen that growth inhibition was already observed at 5 h for all three strains treated with Pinot Noir GSE. This clearly shows the good antimicrobial activity of this extract. Depending on the strain, the degree of inhibition after 5 h was different. It was highest in *S. aureus* and lowest in *E. coli*. At 24 h, it was observed that higher concentrations of the extract inhibited the growth of the strains. The lowest concentration of Pinot Noir GSE that inhibited visible bacterial growth after incubation for 24 h determined the MIC values for each strain. From Figure 2, it can be seen that the strain most sensitive to Pinot Noir GSE was *S. aureus*, and the least sensitive was *E. coli*.

The MIC values of all the GSEs are presented in Table 6. All tested GSEs had significant antimicrobial activity against the investigated bacteria. According to the MIC values, the extracts were ranked in the following order: Pinot Noir > Marselan > Cabernet Sauvignon > Tamyanka. It was found that the extracts were more effective against Gram-positive bacteria than against Gram-negative bacteria. The positive control (ciprofloxacin) exhibited higher antimicrobial activity than the extracts. The MIC against *S. aureus* was 0.04 ± 0.01 mg/mL, the MIC against *B. cereus* was 0.01 ± 0.0 mg/mL, and the MIC against *E. coli* was 0.04 ± 0.01 mg/mL. According to the sensitivity of the strains to the studied extracts, they were ranked in the following order: *S. aureus* > *B. cereus* > *E. coli.*

## 4. Discussion

The study of the antimicrobial and antioxidant activities of natural extracts and their use as alternatives to antimicrobials and food preservatives is a novel and promising endeavor. Grape seed extracts have shown significant antimicrobial activity against various foodborne pathogens [8,17]. The use of GSEs as antimicrobial food additives will allow the replacement of synthetic additives that can have cytotoxic effects [3]. Obtaining these extracts is economically advantageous because they are waste products from the wine industry. The extraction of bioactive compounds from grape seeds for food and medicinal purposes is carried out by various conventional and nonconventional methods, with ethanol as the solvent [17,30]. Studies have indicated that depending on the method, the solvent, and the grape variety, the extraction yield can range from 5 to 25% [12,27,44]. In the present study, extraction yields of 12 to 18% were achieved from the seeds of four grape varieties (Cabernet Sauvignon, Marselan, Pinot Noir, and Tamyanka) using ethanol as the solvent and a conventional method with a magnetic stirrer. When adding extracts to food products, it is very important to preserve the organoleptic properties of the products. Since extracts are introduced at very low concentrations, they do not change the smell, texture, or taste of food products; they can only affect the color. The results of the color intensity tests showed that the extracts of Pinot Noir and Tamyanka had the weakest red color and would not change the color of the products to which they would be applied. 

The resulting extracts are rich in polyphenolic compounds, which give them antioxidant and antimicrobial properties. In order to clarify the antioxidant and antimicrobial potentials of the extracts, it is important to investigate the content of polyphenolic compounds. The data in Table 2 on the indicators in the studied extracts (TPC, TF, TP, TA, and AA) show that they have high values. The TPC of the extracts was in the range of 79.06–111.22 mg GAE/g DW. This corresponds to the results obtained by other authors. Guaita and Bosso (2019) [22] and Castro-Lopeza et al. (2019) [44] reported that in different grape seed extracts, TPC ranged from 7.10 to 107.8 mg GAE/g DW. A study by Sochorova et al. (2020) [21] determined the TPC in the GSEs of 10 grape varieties, and the values were in the range of 8.79–11.27 mg GAE/g. The TF of the extracts was in the range of 40.05–52.01 mg QE/g DW (Table 2). These values are higher than those cited by Guaita and Bosso for GSEs (6.90–25.91 mg QE/g DW) [25] and lower than those cited by Rockenbach et al. (56.01–11.21 mg GAE/g) [25]. The procyanidins in the seed extracts were in the range of 31.43–170.45 mg CE/g DW (Table 2). These results agree with those obtained by other authors for seed extracts of different red grapes (85.2–152.0 mg CE/g DW) [22]. TPC, TF, and TP were found to be the highest in the Pinot Noir and Marselan seed extracts. 

The antioxidant capacities of the extracts were determined by DPPH and ABTS assays. The DPPH assay measures the reducing power of an antioxidant against the DPPH radical. The -OH functional group and its position in the polyphenol structure are responsible for the high scavenging activity of GSEs [45]. The DPPH results, ranging from 245.60 to 579.23 µM TE/g (Table 2), are similar to the results obtained by some other authors [44,46] but higher than those of Rockenbach et al. (2018) [25] for Pinot Noir (169.5 µM TE/g) and Cabernet Sauvignon (82.81 µM TE/g) GSEs. Selkur et al. (2011) [47] determined the antioxidant capacity of Çalkarası grape seeds and showed that the antioxidant activity according to the DPPH method was 9.30 µmol TE/g.

The number of aromatic rings and the molecular weight of polyphenols determine their efficiency in quenching ABTS radicals. [45]. The ABTS results (Table 2) are similar to the results of other studies and show that GSEs are strong ABTS + radical cation inhibitors (1907.24–2273.90 µM TE/g DW). Guaita and Bosso (2019) [22] reported lower ABTS values for seed extracts of different red grape species—109–185 mg/g DW (or 434.91–738.15 µM TE/g DW) but Ghouila et al. (2016) [48] showed higher values—3902 µM TE/g DW. Selkur et al. (2011) [47] reported an antioxidant capacity determined by ABTS of 16.45 µM TE/g. A very good correlation between the TPC results of the studied extracts and their respective antioxidant capacities is evident (Table 2), which shows that the antioxidant parameters were strongly associated with the phenolic values.

An important aspect of studying these extracts is to determine their individual compositions using HPLC. The most frequently detected polyphenolic compound in GSEs is trans-resveratrol. This research shows that the studied extracts were rich in monomeric and dimeric polyphenols (Table 3 and Table 4). Comparing the composition profiles of the four grape varieties showed that the red grape varieties had higher polyphenol content than the white grape variety. The high contents of monomeric phenols ((+)-catechin, 12.16 mg/g DW; (−)-epicatechin, 10.17 mg/g DW) and dimeric polyphenols (procyanidins B_1_, B_2_, B_3_, 15.77 mg/g DW) were striking, especially in the Pinot Noir extract. The results for the same compounds in the Marselan GSE were similar. Cheng et al. (2012) [12] reported similar results, showing that Pinot Noir seed extract contained 14.31 mg/g (+)-catechin and 9.65 mg/g (−)-epicatechin. Most publications have reported lower values of monomeric compounds; for example, in GSE from the red grape Mandilaria, 6.61 mg/g for (+)-catechin and 2.03 mg/g for (−)-epicatechin [49]. In GSE from the red grape Muscat Hamburg, 1.07 mg/g for (+)-catechin and 0.86 mg/g for (−)-epicatechin [50]. Radovanovic et al. (2009) [51] analyzed phenolic compounds using HPLC with GSEs from six grape varieties. Catechin was detected in all GSEs. The highest catechin level was found in Cabernet Sauvignon GSE (0.88 mg/g). Epicatechin was detected only in the GSEs from two varieties, Pinot Noir (0.47 mg/g) and Isabella (−0.18 mg/g).

Composition profile information could help in understanding and improving the antibacterial activity of the extracted agent. In experiments conducted to determine the antimicrobial activity of extracts, it was noted that different bacterial species showed different sensitivities to the extracts. *S. aureus* was the most sensitive, followed by *B. cereus* and *E. coli*. In experiments using the agar diffusion method, the diameters of the growth inhibition zones of *S. aureus*, *B. cereus*, and *E. coli* were found to be 14, 10, and 8 mm, respectively (Figure 2). Radovanovic et al. (2009) [51] reported the same, with growth inhibition diameters of 16 mm for *S. aureus* and 16 mm and 12 mm for *E. coli*. The results of the present study showed that the extracts were more effective against Gram-positive bacteria (*S. aureus* and *B. cereus*) than against Gram-negative bacteria (*E. coli*). Many authors have obtained the same results [17,52,53]. This is due to the presence of a unique liposaccharide cell wall and an efflux pump in Gram-negative organisms [13]. Different types of bacteria require different amounts of time to achieve an inhibitory reaction. During the experiments, it was noted that the extracts started inhibiting growth after 5 h (Figure 2). Baydar et al. (2006) [54] reported that grape seed extract was active against *S. aureus* after 48 h and against *Aeromonas hydrophila* after 1 h.

The results we obtained for the antimicrobial activity of the investigated GSEs against the tested bacteria and the results obtained by other authors are compared in Table 7. Only the data from the uncolored Pinot Noir GSE are shown in Table 7 because it exhibited the highest antimicrobial activity and would not interfere with the color of food products if used as an antimicrobial agent. 

It is obvious that MIC values vary widely in different studies, even for the same microorganism species and the same grape variety. This is due to the different grape varieties, climate, extraction solvents, bacterial species, etc. The comparison shows that the obtained MIC values indicate very good sensitivities of the tested bacteria to the extracts. 

The results indicate that the antimicrobial activity is higher for GSEs from red grape varieties compared to those from white grapes (Table 5 and Table 6). This is due to the richer content of total phenols and monomeric and dimeric phenolic compounds in red grape varieties. Some authors have reported that the antimicrobial activity of seed extracts is mainly due to the rich content of catechin, epicatechin, and dimeric procyanidins [12,31,32,54]. The combination of monomeric, dimeric, and trimeric phenolic compounds provides a complex synergistic antimicrobial effect [17]. Based on the obtained results, some conclusions can be drawn regarding the influence of the total polyphenolic content and composite profile of the studied extracts on their antioxidant and antimicrobial potentials. Comparing our results with those of other authors, it can be seen that the values of TPC and TF and the concentrations of catechin, epicatechin, and procyanidin B1 are comparable and in many cases even higher. The values were highest for the Pinot Noir and Marselan grape extracts. The obtained results show that extracts from these two types of grape seed have the highest antioxidant and antimicrobial activities. The high content of catechin and epicatechin in these seed extracts may be responsible for their high antibacterial activity. However, in order to clearly understand which polyphenol has the greatest inhibitory effect, it is necessary to conduct additional studies in which the polyphenols are isolated and their activities against resistant bacteria are investigated. Based on these data, it will be possible to obtain enriched extracts with higher antioxidant and antimicrobial activities in the future.

## 5. Conclusions

This study partly complements the research in response to the growing demand to create alternative strategies based on natural agents with antimicrobial properties, such as grape seed extracts. GSEs from three red grape varieties, Pinot Noir, Marselan, and Cabernet Sauvignon, and one white grape, Tamyanka, were evaluated. The highest polyphenolic content and antioxidant capacities were recorded for the extracts from the seeds of Pinot Noir and Marselan. The monomeric and dimeric polyphenols in the Pinot Noir and Marselan GSEs were also the highest. These extracts showed the highest antimicrobial activity against three foodborne pathogenic bacteria (*S. aureus*, *B. cereus*, and *E. coli*). Uncolored Pinot Noir GSE is more suitable as a food antimicrobial agent, as its pale color does not change the color of food products. The good antioxidant and antimicrobial activities demonstrated by the obtained GSEs indicate that they have the potential to be applied as antimicrobial agents in food products, thus reducing the allergic risk caused by the use of synthetic preservatives.

## Figures and Tables

**Table 1:** RP-HPLC gradient conditions for grape seed extracts.

Time (min)	Solution A (%)	Solution B (%)
0–5	90	10
5.1–20	80	20
20.1–25	75	25
25.1–50	70	30

**Table 2:** Spectrophotometric determination of phenolic composition and antioxidant capacity of grape seed extracts.

Grapes	TPC (mg GAE/g DW)	TF (mg QE/g DW)	TA (mg CGE/g DW)	AA (mg/g DW)	PC (mg CE/g DW)	DPPH (µM TE/g DW)	ABTS (µM TE/g DW)
Cabernet Sauvignon	88.22 ± 0.72	45.95 ± 0.14	0.05 ± 0.02	3.01 ± 0.11	157.22 ± 2.10	435.25 ± 3.3	2246.23 ± 11.33
Marselan	103.24 ± 1.11	52.01 ± 0.34	0.062 ± 0.01	2.71 ± 0.13	152.18 ± 2.05	597.23 ± 4.12	2273.92 ± 12.32
Pinot Noir	111.22 ± 1.28	51.50 ± 0.30	0.04 ± 0.02	11.07 ± 0.25	170.45 ± 2.52	579.33 ± 4.15	2203.51 ± 10.25
Tamyanka	79.06 ± 0.65	40.05 ± 0.18	no	4.88 ± 0.13	31.44 ± 0.23	245.60 ± 3.23	1907.24 ± 9.56

Bar indicates mean ± SD (*n* = 3).

**Table 3:** HPLC determination of phenolic compounds in grape seed extracts (mg/g DW).

Compound	Pinot Noir	Cabernet Sauvignon	Marselan	Tamyanka
Gallic acid	0.61 ± 0.23	0.44 ± 0.30	0.42 ± 0.31	0.35 ± 0.15
Gallic acid glucoside Procyanidin B_1_	0.88 ± 0.32 8.81 ± 1.09	0.13 ± 0.07 8.82 ± 1.17	0.05 ± 0.01 7.57 ± 0.65	0.64 ± 0.02 7.05 ± 0.73
Procyanidin B_3_	2.90 ± 0.39	0.95 ± 0.37	1.31 ± 0.93	1.69 ± 0.73
(+)-Catechin	12.16 ± 0.98	9.17 ± 0.73	8.06 ± 0.51	7.35 ± 0.50
Procyanidin B_2_	4.06 ± 0.41	5.17 ± 0.21	5.17 ± 0.99	3.46 ± 0.62
(−)-Epicatechin	10.16 ± 1.09	5.94 ± 0.45	14.27 ± 0.64	4.89 ± 0.31
Procyanidin C_1_	0.34 ± 0.11	0.55 ± 0.21	0.68 ± 0.32	0.14 ± 0.07

Bar indicates mean ± SD (*n* = 5).

**Table 4:** Amounts of polyphenolic groups in GSEs, determined by HPLC (mg/g DW).

Group	Compounds	Pinot Noir	Cabernet Sauvignon	Marselan	Tamyanka
Monomeric compounds	(+)-Catechin and (−)-epicatechin	22.32 ± 1.16	15.10 ± 0.916	22.33 ± 1.06	12.24 ± 0.78
Dimeric compounds	Procyanidins B_1_, B_2_, B_3_	15.76 ± 1.07	14.93 ± 1.08	14.05 ± 0.98	12.19 ± 0.67
Trimeric compounds	Procyanidin C_1_	0.34 ± 0.11	0.55 ± 0.21	0.68 ± 0.32	0.136 ± 0.09
Total polyphenols	All polyphenols	38.71 ± 1.72	30.44 ± 1.65	36.87 ± 1.65	24.65 ± 1.52

Bar indicates mean ± SD (*n* = 5).

**Table 5:** Effect of grape seed extracts on the growth of pathogenic microorganisms.

Bacterial Species	Zone of Inhibition (mm)			
	Pinot Noir	Marselan	Cabernet Sauvignon	Tamyanka
*Staphylococcus aureus*	14 ± 0.25	13.5 ± 0.20	9 ± 0.12	8 ± 0.11
*Bacillus cereus*	10 ± 0.15	8.5 ± 0.11	7.5 ± 0.95	7 ± 0.95
*Escherichia coli*	8 ± 0.10	6.5 ± 0.10	-	-

Bar indicates mean ± SD (*n* = 3).

**Table 6:** Minimum inhibitory concentration (MIC) of grape seed extract (GSE) against pathogenic microorganisms.

GSE	MIC (mg/mL)		
	*Staphylococcus aureus*	*Bacillus cereus*	*Escherichia coli*
Pinot Noir	0.12 ± 0.03	0.25 ± 0.08	0.50 ± 0.17
Marselan	0.25 ± 0.04	0.37 ± 0.11	0.50 ± 0.13
Cabernet Sauvignon	0.37 ± 0.07	0.37 ± 0.14	0.75 ± 0.18
Tamyanka	0.50 ± 0.16	0.50 ± 0.17	1.00 ± 0.17

Bar indicates mean ± SD (*n* = 5).

**Table 7:** Comparison of the MICs (mg/mL) of tested GSEs with results from other studies.

GSE	*Bacillus cereus*	*Staphylococcus* *aureus*	*Escherichia coli*	Reference
Black grape	0.05	0.02	0.15	[47]
Commercial GSE, India	10	15	10	[36]
Pinot Noir	-	0.78	25	[13]
Cabernet Sauvignon	-	0.625	-	[48]
Commercial GSE, South Africa	2.5	9.38	12.5	[14]
Pinot Meunier		1.56	100	[13]
Touriga Nacional	0.01	0.1	-	[20]
Karasi	-	5	10	[46]
Commercial GSE, Optipure (Los Angeles, CA, USA)	-	-	4	[49]
Pinot Noir	0.12	0.25	0.50	Current study

## Data Availability

Not applicable.

## Supplementary Materials

The following supporting information can be downloaded at: https://www.mdpi.com/article/10.3390/microorganisms11020395/s1, Figure S1: RP-HPLC analyses of grape seed extract with absorbance detection at 280 nm: GA—gallic acid, GAG—gallic acid glucoside, B1—Procyanidin B1, B3—Procyanidin B3, (+)-C—catechin, B2—Procyanidin B2, (-)-EC—epicatechin, C1—Procyanidin C1. References [24,55] are cited in the supplementary materials.
