# Supplementary material for: Antimicrobial Potential, Antioxidant Activity, and Phenolic Content of Grape Seed Extracts from Four Grape Varieties
Source: Microorganisms. 2023 Feb 3;11(2):395. doi: 10.3390/microorganisms11020395 (PMC9963647; doi:10.3390/microorganisms11020395)
Supplement: Supplementary file 1 [file microorganisms-11-00395-s001.zip › microorganisms-2140121-supplementary.pdf]

Supplementary File

Grape is a phenolic-rich berry with rich antioxidant and antimicrobial properties. Grape seed, skin and pulp are full of different polyphenols associated with healthy benefits [1,2]. In this study, eight compound were detected and quantified in grape seed extracts from four different grape varieties – Pinot Noir, Cabernet Sauvignon, Marselan, Tamyanka. The phenolic composition was determined by reverse phase high performance chromatography (RP-HPLC). Detection was performed by a photo diode array (PDA) detector in the range 200 – 600 nm, and the absorbance was read at 280 nm and 360 nm. The chromatograms at 280 nm were richer and the signal was higher. Consequently, the compound analyses were made at 280 nm (Figure S1).

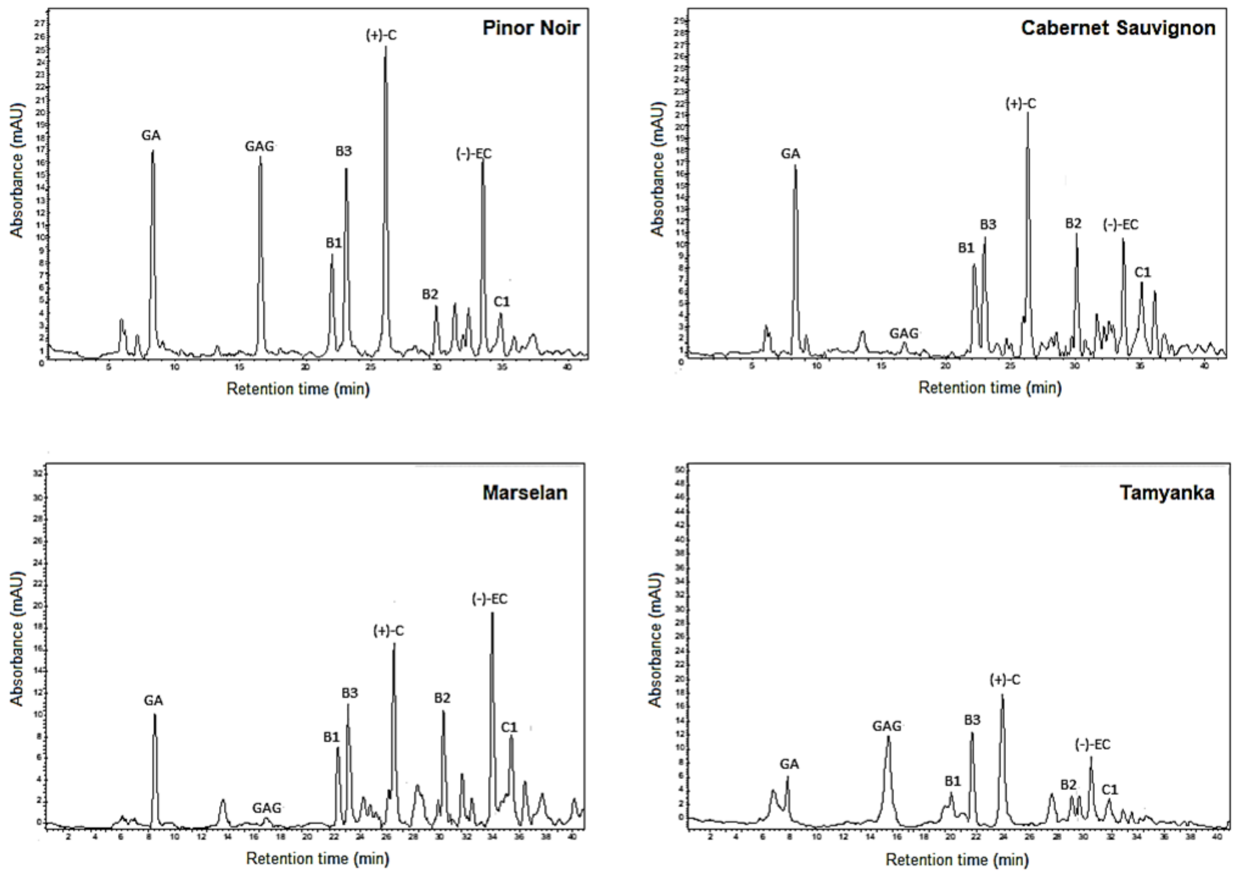

Figure S1. RP-HPLC analyses of grape seed extract with absorbance detection at 280 nm: GA – gallic acid, GAG – gallic acid glucoside, B1 – Procyanidin B1, B3 – Procyanidin B3, (+)-C – catechin, B2 – Procyanidin B2, (-)-EC – epicatechin, C1 – Procyanidin C1.

The sequence of substances leaving the column was established and proven. Analytical standards of the most frequent compounds were used for quantitative correct detection. Eight of them were well expressed in the grape seed samples – gallic acid, gallic acid glucoside (glucogallic acid), (+)-catechin, (-)-epicatechin, Procyanidin B1, Procyanidin B2, Procyanidin B3, Procyanidin C1. The concentration of resveratrol was very low. Epigalocatechin galate, quercetin and rutin were not detected in the extracts. The analyte retention times were proven by adding of a standard to the sample and peak growth observing.

Supplementary References

1. Rajakumari, R., Volova, T., Oluwafemi, O. S., Rajesh Kumar, S., Thomas, S., & Kalarikkal, N. (2020). Grape seed extract-soluplus dispersion and its antioxidant activity. *Drug Development and Industrial Pharmacy*, *46*(8), 1219-1229.
2. Kupe, M., Karatas, N., Unal, M. S., Ercisli, S., Baron, M., & Sochor, J. (2021). Phenolic composition and antioxidant activity of peel, pulp and seed extracts of different clones of the Turkish grape cultivar 'Karaerik'. *Plants*, *10*(10), 2154.
